# New Enzymes as Potential Therapeutic Targets for Trypanosomiases and Leishmaniasis

**DOI:** 10.4061/2011/907423

**Published:** 2012-02-27

**Authors:** Claudio Alejandro Pereira, Ariel Mariano Silber, Elena Gonzalez-Rey

**Affiliations:** ^1^Department of Molecular Biology of Trypanosoma cruzi, Instituto de Investigaciones Medicas Alfredo Lanari (IDIM, UBA-CONICET), Avenida Combatientes de Malvinas 3150, 1427 Buenos Avies, Argentina; ^2^Instituto de Ciencias Biomedicas, Universidade de São Paulo, Av. Lineu Prestes 1374, Cidade Universitária Butanta, São Paulo, CEP 05508-900, Brazil; ^3^Deptartemento Biologia Celular e Inmunologia, Instituto de Parasitologia y Biomedicina “Lopez-Neyra”, Avenida Conocimiento S/N, Parque Tecnologico Ciencias de la Salud, 18100 Armilla, Granada, Spain

Infections caused by the protozoan parasites *Trypanosoma cruzi*, *Trypanosoma brucei*, and *Leishmania* spp. are among the most relevant public health problems in the developing countries. Furthermore, climatic changes and migratory fluxes of the human population are broadening the former geographic restrictions of most of these diseases. Only a few therapeutic treatments are available for these infections, and main drawbacks of drugs in use are their low efficiency, high toxicity, and the emergence of strains resistant to available treatments. All these facts make the research on new drug targets and strategies for developing new drug therapeutic strategies a relevant issue.

In this special issue, we presented original research papers and reviews on the discovery of novel enzyme targets which contributed to having a picture of the state of the art on trypanosomatids' enzymes research oriented to therapeutic applications. The topics addressed in this special edition include a wide variety of enzymes and metabolic pathways: biosynthesis of galactofuranose (M. Oppenheimer et al.), prereplication machinery (S. G. Calderano et al.), glutamate metabolism (A. Magdaleno et al.), ornithine decarboxylases (J. J. Barclay et al.), sphingolipid biosynthetic (C. M. Koeller and N. Heise), phosphotransferase enzymes involved in cell energy management (C. A. Pereira et al.), Heme metabolic pathway (K. E. J. Tripodi et al.), phospholipases A (M. L. Belaunzaran et al.), topoisomerases (B. Banerjee et al.), P4 Nucleases (L. Rahbarnia et al.), enolases (L. Avilan et al.) and in silico prediction of drug targets by non-homologous isofunctional enzymes analysis (M. Rajao Gomes et al.).

I hope the information in this special issue will be useful not only for scientists in the area, but also as a status report on this critical issue for developing countries.

*Claudio Alejandro Pereira* *Claudio Alejandro Pereira* *Ariel Mariano Silber* *Ariel Mariano Silber* *Elena Gonzalez-Rey* *Elena Gonzalez-Rey*

